# Enhancing person-centered obstetric care: A qualitative grounded theory study on perception formation and sense-making during childbirth

**DOI:** 10.18332/ejm/226885

**Published:** 2026-09-04

**Authors:** Mi-Ran Okumu, Lisa Bach, Lissa Haid-Schmallenberg, Ute Karbach, Lorna McKee, Natalie R. Stevens, Anna Volkert, Nadine Scholten

**Affiliations:** 1 Center for Health Services Research and Communication, Department for Psychosomatic Medicine and Psychotherapy, Faculty of Medicine, University Hospital Bonn, Bonn, Germany; 2 Chair of Health Services Research, Institute of Medical Sociology, Health Services Research and Rehabilitation Science, Faculty of Human Sciences and Faculty of Medicine and University Hospital Cologne, University of Cologne, Cologne, Germany; 3 Chair of Quality Development and Evaluation in Rehabilitation, Institute of Medical Sociology, Health Services Research and Rehabilitation Science, Faculty of Human Sciences and Faculty of Medicine and University Hospital Cologne, University of Cologne, Cologne, Germany; 4 Management and Health Services Research, Business School and School of Medicine, Medical Sciences and Nutrition, University of Aberdeen, Aberdeen, Scotland, UK; 5 Lake Cook Behavioral Health, Evanston, USA

**Keywords:** qualitative research, grounded theory, sense of coherence, perinatal care, person-centered care

## Abstract

**INTRODUCTION:**

Quality of obstetric care comprises both provision and experience of care, yet patient experiences have received less attention. Person-centered care seeks to tackle this gap by attributing equal significance to clinical outcomes and care experiences. Individual experiences vary despite similar treatments because subjective appraisals differ. Understanding this process can help to enhance person-centered care in obstetrics. The study’s aim is to explore how birthing persons perceive and make sense of obstetric care experiences.

**METHODS:**

The qualitative study adopted a grounded theory approach according to Corbin and Strauss, utilizing 12 narrative interviews with mothers who gave birth in a hospital a maximum 12 months prior. They were recruited through iterative theoretical sampling through the project Instagram channel between March and August 2023. The iterative process of data collection, analysis and constant comparison resulted in a theoretical model explaining how parturients experience obstetric situations.

**RESULTS:**

During analysis, sense of coherence (SoC) emerged as core category shaping how parturients perceive and make sense of obstetric situations: negative experiences related to low manifestations of SoC and positive experiences to high SoC. A subsequent literature review linked these empirical findings to Antonovsky’s SoC which was contextually adapted to the perception of obstetric situations shaped by comprehensibility, perceived manageability and contextual framing factors.

**CONCLUSIONS:**

Recognizing how parturients construct meaning through their situational SoC, may offer a basis for reflections on interaction with them. Though not allowing for direct generalization, the findings may offer a theoretical concept for further research targeting situational SoC during care processes or piloting feasible measures for real-time assessment.

## INTRODUCTION

Quality of obstetric care encompasses both the provision of care and the experience of care^1^. While evidence-based medicine has made significant advances in the provision of care, patients’ experiences have received comparatively less attention^2^. Person-centered care seeks to bridge this gap by attributing equal significance to clinical outcomes and experiences of care^3^.

Birthing persons experience obstetric situations such as interventions, examinations and decision-making differently^4,5^. While situations appear objectively similar, subjective appraisal may vary, meaning that even medically necessary procedures may be perceived as distressing, coercive or even violent^5,6^. Such experiences may disrupt mothers’ experience of early motherhood, care-giving capacity, and long-term psychosocial well-being^4,5,7^. To better understand these adverse experiences, past research examined this phenomenon under the framework of obstetric violence (OV), exploring it from multiple perspectives, offering definitions, typologies, and theoretical concepts^8-10^. Beyond the specific scope of OV, maternal appraisal of childbirth experiences relates to birth-specific factors comprising the perception of the treatment^11^, perceived control^11-13^, type of interventions carried out^11^, and degree of expectation fulfillment^11,13^, as well as to personal factors such as the demographic and socioeconomic background^11,12^ and prenatal mental health conditions^14^. Apart from these influencing factors, little is known about the interactive perception formation and sense-making process that shape how an obstetric situation is experienced individually. Therefore, the research question of this study was how parturients perceive and make sense of hospital-based care during labor and birth. An understanding of this process may help caregivers to align care with parturients’ subjective needs in the sense of enhancing person-centered care.

## METHODS

### Research setting

The qualitative grounded theory study took place within the BMFTR-funded MAM-Care study (FKZ: 01GY2110) and obtained ethical approval from the Medical Faculty of the University of Cologne (vote no. 22–1260). This work presents parts of the qualitative results in line with the Standards for Reporting Qualitative Research (SRQR)^15^ and the Guideline for Reporting and Evaluating Grounded Theory Research Studies (GUREGT)^16^ (Supplementary file).

### Study design and theoretical framework

Focusing on the subjective perception formation of parturients, we took a grounded theory approach according to Corbin and Stauss^17^. Grounded theory is a methodology of symbolic interactionism in which social order is viewed as an ongoing process of interaction and interpretation^18^. To explain social phenomena, the actor’s perspective or definition of the situation needs to be reconstructed and understood.

Targeted literature searches were conducted at the onset, during analysis, and after identification of the core category. At the onset, sensitizing concepts^19^ were guided by dimensions of childbirth experiences in existing literature such as perceived level of control, interpersonal relationship with caregivers, as well as theories of perception and cognitive appraisal^20-22^. According to the latter, the outside world is understood and evaluated by the individual’s inner world. A subsequent targeted literature review linked the empirical findings to the concept of sense of coherence (SoC) of Antonovsky^23^, which was contextually adapted to the study and research question.

### Participants and recruitment

Data collection comprised narrative interviews with mothers who fulfilled the eligibility criteria, including a hospital birth no longer than 12 months prior to data collection. The specific time frame was chosen so that participants’ memories of the birth experience were still fresh, thereby minimizing recall bias. Recruitment took place through the MAM-Care Instagram channel (Supplementary file) in March, July and August 2023. Participants received an allowance of 50€. In total, 17 mothers responded to our calls, of which five did not meet the inclusion criteria. The sample comprised 12 mothers (mean age 34 years, range 27–41 years) who were predominantly primiparous and had a vaginal delivery, with varying levels of education and health insurance status.

The recruitment strategy followed an iterative theoretical sampling approach. In order to maximize the discovery of categories during initial sampling, the first and second posts intentionally called for extreme cases, ‘Obstetric violence: Breaking the taboo – talking about it’. To capture participants’ subjective perceptions, the term OV was deliberately left undefined. Despite this recruitment framing, participants described a full spectrum of birth experiences ranging from distressing or violent to positive ones (see Data collection). Within the first interviews, fundal pressure was mentioned as a negative and violent experience by two interviewees. This intervention involves manual pressure to the uppermost part of the uterus towards the birth canal^24^. A literature search revealed controversial parturient experiences of fundal pressure varying from supportive and positive to violent and traumatic^25^. The third call addressed persons who had experienced fundal pressure. To capture a variety of experiences, in the Instagram post the intervention was described neutrally without reference to OV. This study deals with all aspects of the interviews except the experience of fundal pressure, which is addressed in a separate publication^26^.

### Data collection

The interviews were primarily inductive and included narrative prompts (Supplementary file). The first narrative prompt referred to the recruitment topic (experience of OV or fundal pressure). To explore the entire birth experience, narrative prompts also addressed further experiences in the delivery room as well as prior expectations. After each narration, follow-up questions were asked. Participants were informed about the possibility of interrupting or ending the interview at any time if experiencing distress. Participants showing signs of distress related to the childbirth experience were offered resources of information and counseling. Given the study’s focus on the participants’ perspective, clinical documentation of complications or interventions was not collected. Instead, participants voluntarily described relevant clinical events during the interviews, which were captured as preceding conditions to the respective situation.

The interviews were conducted in person in the project office or via video call by the first author (MO) in 2023. The interviews of the first call were conducted in March and May. The second call included an interview in July. The interviews of the third call were conducted in August. Prior to the interviews, MRO conducted phone calls to introduce herself, explain the procedure, and answer questions. All participants gave their written consent prior to the interview. The duration of the interviews ranged from 31 to 116 minutes. All interviews were audio-recorded, transcribed and anonymised^27^. During and/or after the interviews, handwritten field notes were taken, which were subsequently integrated into the analysis.

### Analysis

Our approach involved circular data collection, analysis and theory formation until reaching theoretical saturation when no new topics emerged, and the categories and their interrelations were elaborated and validated. We conducted sequential analysis and applied constant comparison to inductively derive concepts and categories. Concepts were contrasted with existing literature. Analysis involved open, axial, and selective coding^28^. The first interviews were coded line-by-line. During analysis, each interview transcript was maintained in a separate working document (Microsoft Word and Excel) with the text divided into analytical segments. These were annotated with memos, paraphrases, initial coding ideas, and reflections of theoretical relationships. During axial coding, we used the coding paradigm^28^ to systematically reflect the relationships among the emerged categories. The central category emerged and was solidified during the selective coding phase. The emerging theoretical concept was continuously verified against the data through constant comparison, memoing, and theoretical sampling.

The analysis was mainly conducted by three female project researchers (LB, LHS, MRO), with backgrounds in education, sociology and midwifery. LHS works in perinatal care while LB and MRO are mothers who gave birth in hospitals. Interpretative disagreements were reconciled in iterative dialogues and constant comparative analysis. To mitigate potential interpretative influence and enhance intersubjective comprehensibility, apart from regular reflective discussions among the coders, the findings were also discussed with the wider project team and a qualitative research circle.

## RESULTS

In total, 12 interviews were conducted. Table 1 presents the characteristics of our 12 participants (P1–P12) who all identified as female.

**Table 1:** Characteristics of participants, interviewed in person or via video call, March–August 2023 (N=12, theoretical sampling)

*Characteristics*	*n*
**Age at the time of delivery** (years), mean (range)	34 (27–41)
**Mode of delivery**	
Vaginal	8
Instrumental vaginal	2
Secondary cesarean section	2
**Parity**	
Primiparous	11
Multiparous	1
**Education level**	
Lower than A-levels	1
A-levels	3
University	8
**Health insurance**	
Statutory	9
Private	3

**Figure 1:**
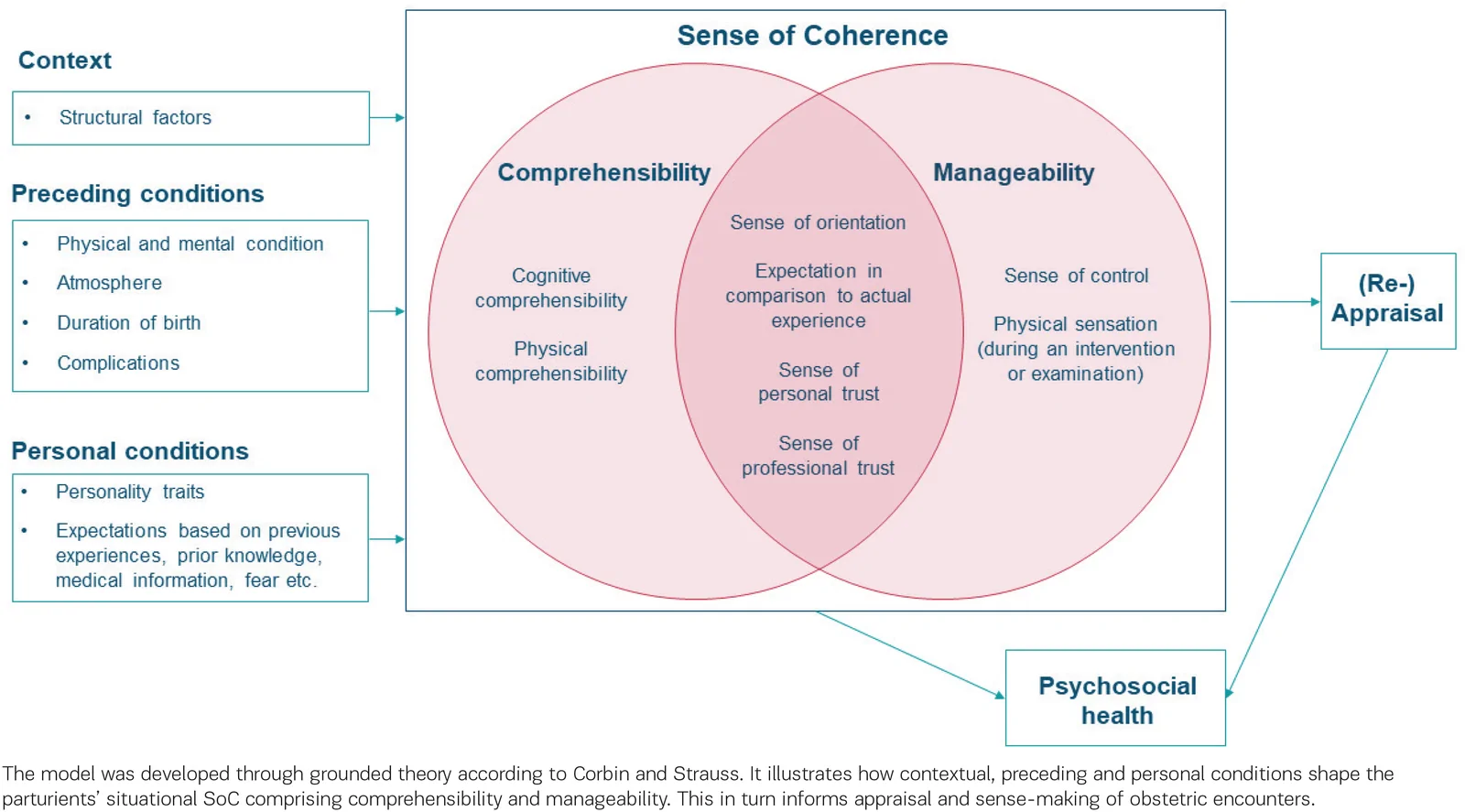
Theoretical model on perception formation and sense-making regarding obstetric situations

One participant reported full satisfaction with her birth experience, while all other participants described both positive and negative or violent experiences. Based on the findings, we developed a conceptual model (Figure 1) explaining the process by which obstetric situations are perceived. An overview of the category system is given in Table 2.

**Table 2:** Category system from interviews of participants, in person or via video call, March–August 2023 (N=12, theoretical sampling)

*Category*	*Sub-categories*	*Representative quotes*
Comprehensibility	Cognitive comprehensibility	*‘Then she said … that I should lie on my back, but I had often heard that this is not good and that you should try out different positions first.’* (P1)
	Physical comprehensibility	*‘Somehow, I always closed my legs automatically. And then she always told me not to do that. But … this was the movement my body wanted to make when the contraction came. And she somehow deprived me of that, I didn’t find that very encouraging.’* (P5)
Manageability	Sense of control	*‘Then they explained it [how to push] to me, and it was really helpful, the way they guided me through the birth process … they explained until I understood how it all worked and how my body was reacting. You need to develop a sense for it first – what to do during a contraction, what feels right and what doesn’t. You have to get a feel for the situation. But once I mastered that, I truly felt like it was my delivery room, and that really helped.’* (P4)
	Physical sensation during an intervention or examination	*‘I kept explaining that I was in pain, that I could feel everything [epidural not effective] … examinations were carried out regarding the dilation of the cervix grasping and stretching with full brutality … always under the assumption that I had a functioning epidural.’* (P2)
Overlap of comprehensibility and manageability	Sense of orientation	*‘There was always someone there. I mean, of course there wasn’t always someone standing right there in the room, that’s not possible, but when you rang the bell, someone always came … You didn’t have to wait long for someone to arrive. So, there was always someone there, that was very positive.’* (P6)
	Expectation in comparison to actual experience	*‘I didn’t feel like going into the delivery room, I personally didn’t find the atmosphere in the labor ward as relaxing as I had imagined.’* (P11)
	Sense of personal trust	*‘I would have liked her [midwife] to believe me and that she would have responded to my wishes and that she would not show me that she was annoyed. Because that was really horrible for me … I would have rather needed someone who says: “Yes, you’re doing great”.’* (P1)
	Sense of professional trust	*‘There was a midwife who, in my opinion, was totally overwhelmed, she kept looking at me as if I was to tell her what to do next … so I didn’t feel comfortable with her at all.’* (P10)
Context	Structural factors	*‘Actually, the obstetrician was very empathetic and kind, just like the midwife. I just thought that they weren’t in the room much. And with the knowledge that there was no central monitoring, this created uncertainty.’* (P3)
Personal conditions	Personality traits	*‘I’m the kind of person who would like to know what’s coming next or what else I can expect.’* (P7)
	Expectations (based on previous experiences, prior knowledge, medical information, fear, etc.)	*‘I was extremely nervous right from the start and I was also terrified … That’s why I decided to have an epidural, because I was terrified of the pain.’* (P2)
Preceding conditions	Physical and mental condition	*‘And this anesthetist informed me about the risks of the epidural in a state where I would have signed anything. I can’t remember the conversation. I don’t even know what my signature looked like on this sheet.’* (P10)
	Atmosphere	*‘I really would have liked a bit more of a feel-good atmosphere. Unfortunately, as everywhere in hospitals, they were simply very thinly staffed and were just very hectic and focused on getting on with work … For you, it’s a once-in-a-lifetime momentum and for them it’s just one of hundreds of births* … *So, nobody ever asked: “Is that okay for you? Or do you need something else?”.’* (P11)
	Duration of birth	*‘I had … the feeling that I no longer had the strength after such a long time … I think I was in labor for 22 hours … and awake for over 26 hours.’* (P3)
	Complications	*‘I was really under a lot of time pressure the whole time and it was always between “You have to co-operate, the fetal heart rate is getting worse. You shouldn’t be panicking now, relax, but please cooperate”, and that was really such a psychological stress at that moment.’* (P1)

* Categories and sub-categories emerged inductively through iterative open, axial and selective coding, constant comparison and systematic application of the coding paradigm until reaching theoretical saturation.

Within our sample, the negative experiences were closely related to a sense of incoherence, while positive experiences were related to a sense of coherence. The sense of coherence emerged as a core category. A subsequent literature research revealed parallels to the SoC of Antonovsky^23^ reflecting a global life orientation and coping capacity. We adapted the concept to align with our research context and findings.

### Comprehensibility

Participants emphasized the importance of comprehension. Comprehensibility was formed intrinsically and/or extrinsically. The latter was based on the communication of caregivers. Participants described how cognitive comprehensibility lowered feelings of vulnerability:

*‘They [caregivers] explained a lot, which was always good for me, to understand things.’* (P12)

In contrast, low levels of comprehensibility were perceived negatively:

*‘I felt quite uncomfortable because I didn’t know what was going to happen to me.’* (P6)

Participants mentioned instances where their physical intuition (physical comprehensibility) influenced their cognitive understanding. A participant described how the midwife’s instructions against her intuition were incomprehensible to her:

*‘I kept saying that it [all fours] wasn’t working. It felt as if something, the baby, was stuck.’* (P1)

### Manageability

Participants described their perceived ability to cope with situations (manageability). While a high sense of control was perceived as reassuring and empowering, participants linked a low sense of control with lack of self-determination and self-efficacy. A participant’s epidural was not effective, but she was not believed:

*‘I was just completely helpless and completely at their mercy because I wasn’t taken seriously at all.’* (P2)

Participants’ perception was further influenced by the physical sensation during an intervention or examination. Particularly feeling sudden strong pain through interventions or examinations was associated with violence and was differentiated from continuous labor pain:

*‘They catheterized me quite often to drain my urine* … *that hurt like hell.’* (P2)

### Overlap of comprehensibility and manageability

Certain aspects mentioned by the participants influenced both comprehensibility and manageability. Participants referred to a sense of orientation related to caregivers who accompanied the process:

*‘I just felt very much left alone at that moment because no one was there* … *What can I do now? Can I walk around?* … *At some point I didn’t even dare to stand up.’* (P10)

A participant described how hospital staff managed to convey a sense of orientation despite standard routines and shift changes:

*‘They always introduced themselves, explaining their positions and how long they would be with me. They always said goodbye at the end of their shift when new staff arrived, letting me know who was coming next. I really appreciated that.’* (P7)

Participants further assessed their expectations in comparison to the actual experience. The expectations were based on explanations of caregivers, prior knowledge, experiences, and prevailing societal opinions, among others:

*‘… what I would actually have liked. To be able to move much more and not be so attached to equipment and cables. That was really annoying …’* (P12)

Trust was mentioned as a crucial factor. Participants emphasized the importance of feeling understood, reassured, accepted, and taken seriously (interpersonal trust). A participant was in pain for 12 hours before an anesthetist finally confirmed that the epidural did not function:

*‘They could have ended this suffering after just one hour if they had realized that they had made a mistake and had replaced the epidural. Or if they had simply believed me* …*’* (P2)

In contrast, a participant experienced a high level of interpersonal trust:

*‘I’m a medical layperson* … *but I was asked for my opinion* … *“this is your body, and you know yourself best”.’* (P4)

Participants also referred to the perceived expertise of the hospital staff (professional trust):

*‘I followed the recommendations, but I thought they were rubbish.’* (P1)

### Sense of coherence (SoC)

Positive experiences were linked to high SoC while negative experiences were linked to low SoC. Individual relevance setting also played a role, e.g. some participants highlighted the pain felt during an intervention while others emphasized aspects such as feeling patronized. Table 3 presents two vaginal examination experiences, one perceived as violent with low comprehensibility and manageability, and one with higher levels.

**Table 3:** Conceptual contrast within the core category SoC exemplified at divergent experiences of vaginal examinations by two participants, from interviews in person or via video call, March–August 2023 (N=12, theoretical sampling)

*Emergent categories*	*Participant 9 Negative perceptions*	*Participant 12 Positive perceptions*
**Sense of comprehensibility**	*‘When I was lying in the labor room bed, the midwife who was on duty at the time examined me vaginally without telling me beforehand.’*	*‘I always had the feeling, ‘okay, she‘s not doing this [vaginal examination] out of boredom, but it‘s contributing to the progress of my birth.’*
**Sense of manageability**	*‘She examined my cervix without saying anything and I thought that was totally intrusive.’* *‘I just suddenly realized there was something there and it hurt like crazy. I also remember that I totally cried out loud.’*	*‘It [vaginal examination] was unpleasant … But it wasn‘t that bad … maybe it was because of how the staff did it and how they explained it, because whenever they did it, they wouldn‘t just say: “I‘m doing it”. They didn‘t just do it, but they always told me somehow: “Listen, we‘ll have another look here now, because”.’*
**Comparative interpretation**	The participant was taken by surprise by an unannounced vaginal examination and described lack of comprehensibility, strong pain and lack of control.	The participant comprehended why the vaginal examination was necessary and experienced interpersonal trust and control despite the examination being unpleasant.

SoC: sense of coherence.

Interviewees described comprehensible situations that felt not manageable as harmful or negative. Participant 10 understood the situation yet experienced it as hardly bearable:

*‘*… *every day they said, “We will induce labor tomorrow* …*”. They kept postponing the induction because so many children were born* … *There was simply not enough staff. That was really horrible for me.’* (P10)

Participant 4 felt lost and stressed by the start of the second stage of labor. After the midwife answered her questions and offered appropriate support, she regained her high level of SoC.

### (Re-)Appraisal

Appraisals took place in the situation and/or shortly afterward:

*‘I experienced it as so violent, especially the emotional part. That really affected me.’* (P1)

In some cases, a re-appraisal took place after a while. In this regard, participants mentioned the wish for debriefings with hospital staff to get explanations and make sense of what happened and why.

### Framing factors

Within our sample, the formation of the SoC was framed by general factors which are outlined below.

### Context

Participants mentioned structural factors such as infrastructure, organization, staffing levels, general rules and procedures as influencing their birthing experience:

*‘*… *there was simply not enough staff, even after birth.* … *no one was there to suture me. There was no one to remove the epidural.’* (P10)

### Personal conditions

Participants’ anticipations of situations were based on communication from caregivers, prior knowledge, and emotions. We subsumed these aspects as expectations. Under personality traits, we outlined patterns of thoughts, feelings and behaviors:

*‘I’m generally sensitive, but of course that was a* … *day, where you’re somehow even more sensitive.’* (P1)

### Preceding conditions

Participants described factors preceding the experiences, such as the labor ward atmosphere (e.g. hectic, calm). Further, the duration of birth as well as complications were mentioned. Several participants referred to their physical and mental condition, highlighting their cognitive impairment due to continuous labor pain and exhaustion.

## DISCUSSION

This study aimed to deepen the understanding of birthing persons’ perception and sense-making process of obstetric situations. Within our sample, situational SoC comprising comprehensibility (understanding of what is happening) and manageability (ability to cope with the situation) emerged as a central influencing factor. Our findings may indicate that situational SoC is formed recurrently during childbirth. The various manifestations of SoC in different situations raise the possibility that both SoC and its influencing factors (sub-categories) may be continuums. Our findings potentially indicate a close interrelation between comprehensibility and manageability. The level of comprehensibility could influence the perceived manageability, e.g. uncomfortable examinations became more bearable if comprehensible. Situations that were comprehensible but not manageable were experienced negatively. This may indicate that the perceived ability to cope with an obstetric situation is the decisive aspect regarding the appraisal of an experience and leads to the interpretation that comprehensibility may affect manageability while manageability may predominantly determine the appraisal of an experience.

The concept of SoC goes back to Antonovsky^23^ who saw high manageability as contingent on comprehensibility, while in our context a lower comprehensibility may be more bearable due to the conventional roles of medical caregivers and patients. Antonovsky considered meaningfulness as the most important component^29^. Drawing on our findings, we suggest that in obstetric experiences, comprehensibility and particularly manageability appear to be salient aspects while meaningfulness seems to emerge through (re-)appraisal. Past studies have shown the relevance of SoC regarding the attitude towards labor and birth or the occurrence of complications^30^ and measured SoC during pregnancy and/or postpartum^31,32^. Our findings do not contradict the model of Antonovsky^23^ but contextualize and extend it by framing SoC as a dynamic, continuous process of perception formation and sense-making during childbirth. While the Antonovsky^23^ SoC reflects a person’s view of life and capacity to respond to stressful situations, our grounded theory study focuses on situation-specific SoC during childbirth and obstetric situations.

Furthermore, our findings align with the transactional model of stress and coping of Lazarus and Folkman^21,22^, which depicts three types of appraisals regarding potential stressors: primary (assessing the situation), secondary (assessing the available coping resources), and re-appraisal based on new information. From our analysis, comprehensibility mirrors primary appraisal, while manageability included aspects of coping with the situation. Further, some participants reported re-appraisals.

Our categories correspond with previous research associating experience of OV with lack of cognitive^4^ and physical^7^ comprehensibility, perceived control^4,7,12^, physical pain^7,9^, interpersonal relations^4,11^ and fulfillment of expectations^4,13^. Long birth durations, complications and subsequent interventions^9,11^ were shown to potentially increase vulnerability, but how these were perceived was related to the care and communication of caregivers^4^.

The results tentatively indicate that the perception of comprehensibility and manageability strongly relates to how parturients experience the interaction of hospital staff. This would imply that caregivers can actively influence the level of SoC. This aligns with prior studies highlighting that caregivers’ communication may strengthen birthing persons’ SoC^4^. At the same time, it was also revealed that the interaction may easily be challenged by misconceptions and different perceptions^33^. This leads to the interpretation that the same intervention or examination may be experienced differently by different persons. According to symbolic interactionism, interaction is a reciprocal and co-constructive process^18^. Therefore, the parturient’s situational SoC may likely shape the interaction with caregivers and vice versa. However, given the study’s focus on parturients, the caregivers’ perspective of SoC was not examined.

Based on our findings, we suggest that continuously assessing the situational SoC and taking appropriate measures to enhance it can strengthen person-centered care. Caregivers should be aware of individual needs and preferences such as varying levels of desire for control^34^ and acknowledge that some parturients may be more vulnerable, e.g. persons with psychiatric history, antenatal distress, complications during childbirth or primiparous parturients^35^. Such factors, along with individual preferences and dislikes, could be documented during prenatal appointments and complemented by careful observation and communication during childbirth. Due to the dynamic nature of birth processes, the situational SoC may need to be assessed continuously during birth. The Artinian Intersystem Model (AIM)-care plan^36^ offers a comprehensive framework for tracking comprehensibility, manageability, and meaningfulness alongside physiological or psychosocial aspects, among others. However, such a comprehensive assessment may be too complex for birth situations. A feasible alternative would need to allow a quick and simple real-time assessment of comprehensibility and manageability, e.g. through observational cues or short verbal check-ins.

### Strength and limitations

Although participants had individually distinct experiences with different staff in different hospitals, they addressed the same topics. A strength of the study lies in the rich, context-specific insights which converge with the project’s quantitative data. Despite the highly sensitive nature of the topic and the challenge of recruiting mothers in their first year postpartum, we received sufficient participation to achieve theoretical saturation. Although retrospective narratives inherently carry potential for recall and social desirability bias, participants largely distinguished between immediate perceptions in the actual situation and later reflections. Later re-appraisals were framed as such. Nevertheless, the temporal distance from the birth experience may have limited the accuracy of recalling situational interactions and perceptions. The sample was predominantly primiparous and highly educated, likely reflecting the reach of Instagram-based recruitment. We acknowledge that participants in this sample were likely well prepared through antenatal classes and available information. However, our findings revealed that even highly prepared participants could encounter situations that were unexpected and diverged from their expectations. This may highlight that the situational SoC is shaped both by preceding, contextual, and personal conditions but also emerges dynamically in the situation. During recruitment, the financial incentives seemingly played a subordinate role, as most participants emphasized that their participation was driven by personal relevance of the topic and the desire to improve maternity care. While the homogeneity of the sample demonstrates boundaries of our findings, future studies should include more diversity in terms of parity, educational, ethnic, and socioeconomic backgrounds.

## CONCLUSIONS

Recognizing how parturients construct meaning during the birth process through comprehensibility and manageability may offer caregivers a basis to reflect on their interaction with them. While these qualitative findings do not allow for generalization, they may offer a theoretical concept for further research. Future studies could examine parturients’ situational SoC during the care process or could pilot feasible measures to assess the situational SoC in real time during various situations of childbirth and triangulate subjective experiences of parturients with clinical documentation.

## Supplementary Material



## Data Availability

Due to the study’s sensitive nature and the assurance of anonymity, the data are not publicly available.
