# Supplementary material for: Enhancing person-centered obstetric care: A qualitative grounded theory study on perception formation and sense-making during childbirth
Source: Eur J Midwifery. 2026 Sep 4;10:10.18332/ejm/226885. doi: 10.18332/ejm/226885 (PMC13544188; doi:10.18332/ejm/226885)
Supplement: Supplementary file 1 [file EJM-10-41-s1.pdf]

EUROPEAN JOURNAL
OF MIDWIFERY

**Supplementary file**

© 2026 Okumu M. R. et al.

**DOI:**
10.18332/ejm/226885

The content has been provided by the author(s) and has not been reviewed, verified, or endorsed by European Publishing. It may not have undergone peer review. The views, opinions, and recommendations expressed are solely those of the author(s) and do not necessarily reflect the position of European Publishing. European Publishing accepts no responsibility or liability for any consequences arising from the use of, or reliance on, this content.

**Standards for Reporting Qualitative Research (SRQR)**

| | |
|---|---|
| **Title and abstract** | |
| **Title** – Concise description of the nature and topic of the study identifying the study as qualitative or indicating the approach (e.g., ethnography, grounded theory) or data collection methods (e.g., interview, focus group) is recommended | Title page |
| **Abstract** – Summary of key elements of the study using the abstract format of the intended publication; typically includes background, purpose, methods, results, and conclusions | Abstract page |
| **Introduction** | |
| **Problem formulation** – Description and significance of the problem/phenomenon studied; review of relevant theory and empirical work; problem statement | Lines 2-20 |
| **Purpose or research question** – Purpose of the study and specific objectives or questions | Lines 20-23 |
| **Methods** | |
| **Qualitative approach and research paradigm** – Qualitative approach (e.g., ethnography, grounded theory, case study, phenomenology, narrative research) and guiding theory if appropriate; identifying the research paradigm (e.g., postpositivist, constructivist/interpretivist) is also recommended; rationale | Lines 34-38 |
| **Researcher characteristics and reflexivity** – Researcher's characteristics that may influence the research, including personal attributes, qualifications/experience, relationship with participants, assumptions, and/or presuppositions; potential or actual interaction between researchers' characteristics and the research questions, approach, methods, results, and/or transferability | Lines 109-111 |
| **Context** – Setting/site and salient contextual factors; rationale | Lines 27-29, 86 |
| **Sampling strategy** – How and why research participants, documents, or events were selected; criteria for deciding when no further sampling was necessary (e.g., sampling saturation; rationale | Lines 49-70 |
| **Ethical issues pertaining to human subjects** – Documentation of approval by an appropriate ethics review board and participant consent, or explanation for lack thereof; other confidentiality and data security issues | Lines 27-29 |
| **Data collection methods** – Types of data collected; details of data collection procedures including (as appropriate) start and stop dates of data collection and analysis, iterative process, triangulations of sources/methods, and modification of procedures in response to evolving study findings; rationale | Lines 74-93 |
| **Data collection instruments and technologies** – Description of instruments (e.g., interview guides, questionnaires) and devices (e.g. audio recorders) used for data collection; if/how the instrument(s) changed over the course of the study | Lines 74-93 |
| **Units of study** – Number and relevant characteristics of participants, documents, or events included in the study; level of participation (could be reported in results) | Lines 54-57, Table 1 |
| **Data processing** – Methods for processing data prior to and during analysis, including transcription, data entry, data management and security, verification of data integrity, data coding, and anonymization/deidentification of excerpts | Lines 91-93 |
| **Data analysis** – Process by which inferences, themes, etc., were identified and developed, including the researchers involved in data analysis; usually references a specific paradigm or approach; rationale | Lines 96-108 |
| **Techniques to enhance trustworthiness** – Techniques to enhance trustworthiness and credibility of data analysis (e.g., member checking, audit trail, triangulation); rationale | Lines 73-75 |
| **Results/findings** | |
| **Synthesis and interpretation** – Main findings (e.g. interpretations, inferences, and themes); might include development of a theory or model, or integration with prior research or theory | Lines 118-224, Table 2, Table 3, Figure 1 |
| **Links to empirical data** – Evidence (e.g. quotes, field notes, text excerpts, photographs) to substantiate analytic findings | Lines 118-224, Table 2, Table 3 |
| **Discussion** | |

| | |
|---|---|
| **Integration with prior work, implications, transferability, and contribution(s) to the field** – Short summary of main findings; explanation of how findings and conclusions connect to, support, elaborate on, or challenge conclusions of earlier scholarship; discussion of scope of application/generalizability; identification of unique contribution(s) to scholarship in a discipline or field | Lines 227-291, 316-322 |
| **Limitations** – Trustworthiness and limitations of findings | Lines 294-313 |
| **Other** | |
| **Conflicts of interest** – Potential sources of influence or perceived influence on study conduct and conclusions; how these were managed | Declaration of interest |
| **Funding** – Sources of funding and other support; role of funders in data collection, interpretation, and reporting | Funding statement |

**The Guideline for Reporting and Evaluating Grounded Theory research studies (GUREGT) (Berthelsen, Grimshaw-Aagaard, Hansen 2018)**

| Main area | Item | Grounded theory methodology: Strauss and Corbin | Lines |
|---|---|---|---|
| Study aim | 1 | Is the grounded theory study aim presented to develop a well-integrated set of concepts that provide a theoretical explanation of a social phenomenon? | 18-22 |
| Philosophical framework | 2 | How is the grounded theory embedded in symbolic interactionism? | 35-38 |
| The researcher's role | 3 | Is the researchers theoretical sensitivity according to theoretical insight, professional and personal experience, described and explained? | 33-46, 105-108 |
| Data collection | 4 | Is data collection methods described and explained? | 73-84 |
| | 5 | Have qualitative or quantitative data collection methods been used? How and why? | 74-78 |
| Memos | 6 | Have field notes and diagramming been written and used throughout the study about concepts and categories and are they used to formulate and develop the theory? | 92-93, 101-104 |
| Sampling procedures | 7 | Is open, relational and variational sampling conducted in the beginning of data collection described and explained? | 58-70 |
| | 8 | Is theoretical and discriminate sampling of the emerging categories and theory from the data collection described and explained? | 96-99 |
| | 9 | Is the selection of participants guided by theoretical sampling? How? | 58 |
| Theoretical saturation | 10 | Is the reach of theoretical saturation explained according to new insights relevant for the emergent theory? | 96-98 |
| Analysis and coding | 11 | Is the coding levels and concurrent process of coding described according to open, axial (the paradigm model) and selective coding? And is matrix building and storyline applied and described? How? | 100-105 |
| | 12 | Which categories have guided the specific coding levels and how? | 127-129 |
| | 13 | Is the central category identified before conducting selective coding? | 105-106 |
| | 14 | Which categories have contributed to identify the density, internal consistency and gaps in logic of the parsimonious theory? | 100-104 |
| | 15 | Is the constant comparison method used to compare incidents with incidents, incidents with categories and categories with categories? | 98-99 |
| | 16 | Is the simultaneous data collection, analysis and coding guided by the theoretical sampling and writing memos described and explained? | 96-104 |
| Review of literature | 17 | Is general literature reviewed initially in the grounded theory study to assist in formulating questions? Why and how? | 39-40 |
| | 18 | Is an extensive literature review performed after theory development on the basis of the emerging concepts and theory? How and on what grounds? | 39-46, 128-131 |
| Results/the theory | 19 | Is the main concern presented and explained? | Figure 1 |
| | 20 | Are the central category and the related categories, properties and dimensions presented and described? | 127-129, Table 2, Table 3 |
| | 21 | Does the theory provide a thourough theoretical explanation of the social phenomenon? | Figure 1, 227-240 |
| | 22 | Are quotes used and argued for to describe the theory? | 123-224, Table 1, Table 2 |
| Discussion | 23 | Are the key relationships between the central category and categories discussed and related to relevant literature? | 227-291 |
| Evaluation criteria | 24 | Are the criteria of validity, reliability and credibility of data presented and explained? | 106-108 |
| | 25 | Are the evaluation criteria used to evaluate the theory? | 106-108 |

# OBSTETRIC VIOLENCE
## *Interview participants sought*

BREAKING THE TABOO - TALKING ABOUT IT

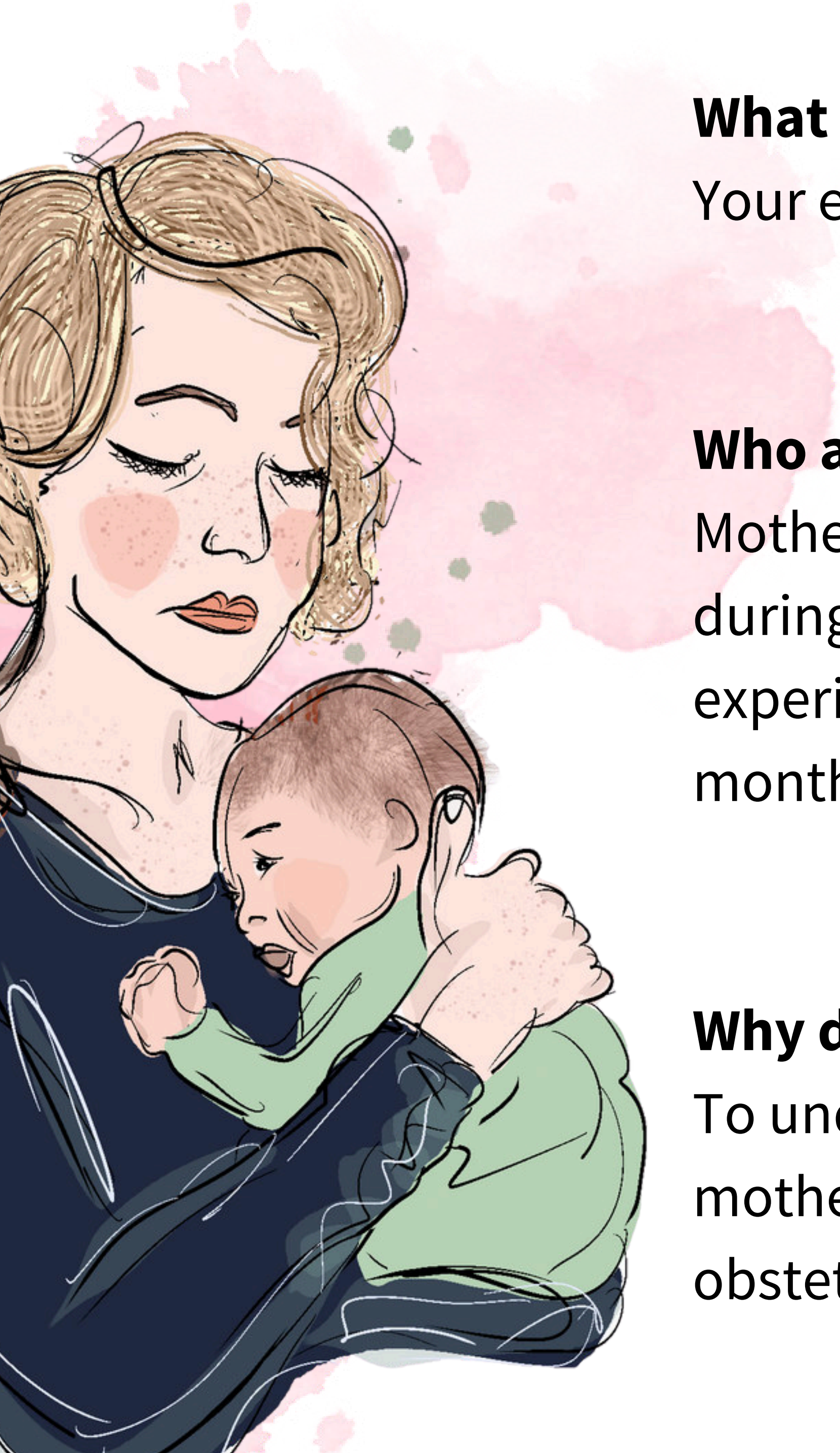

**What is this about?**
Your experiences of violence during labour and childbirth

**Who are we looking for?**
Mothers who experienced obstetric violence of any kind during the birth of their child in the hospital. The birth experience should not have taken place more than 12 months ago.

**Why do we ask about this?**
To understand the background of obstetric violence from mothers' perspectives and improve hospital based obstetric care in the long term.

# Experienced fundal pressure / Kristeller maneuver*?

## Interview participants sought

**What is it about?**

*Fundal pressure means pressure on the upper abdomen applied by hospital staff during labour.

**Who are we looking for?**

Mothers who experienced this intervention during labour and would like to share their experience with us in an interview. The birth experience should not have taken place more than **12 Months** ago.

**Why do we ask about this?**

Obstetric measures are experienced very differently. In order to improve obstetric care for the future, we would like to better understand your perspective.

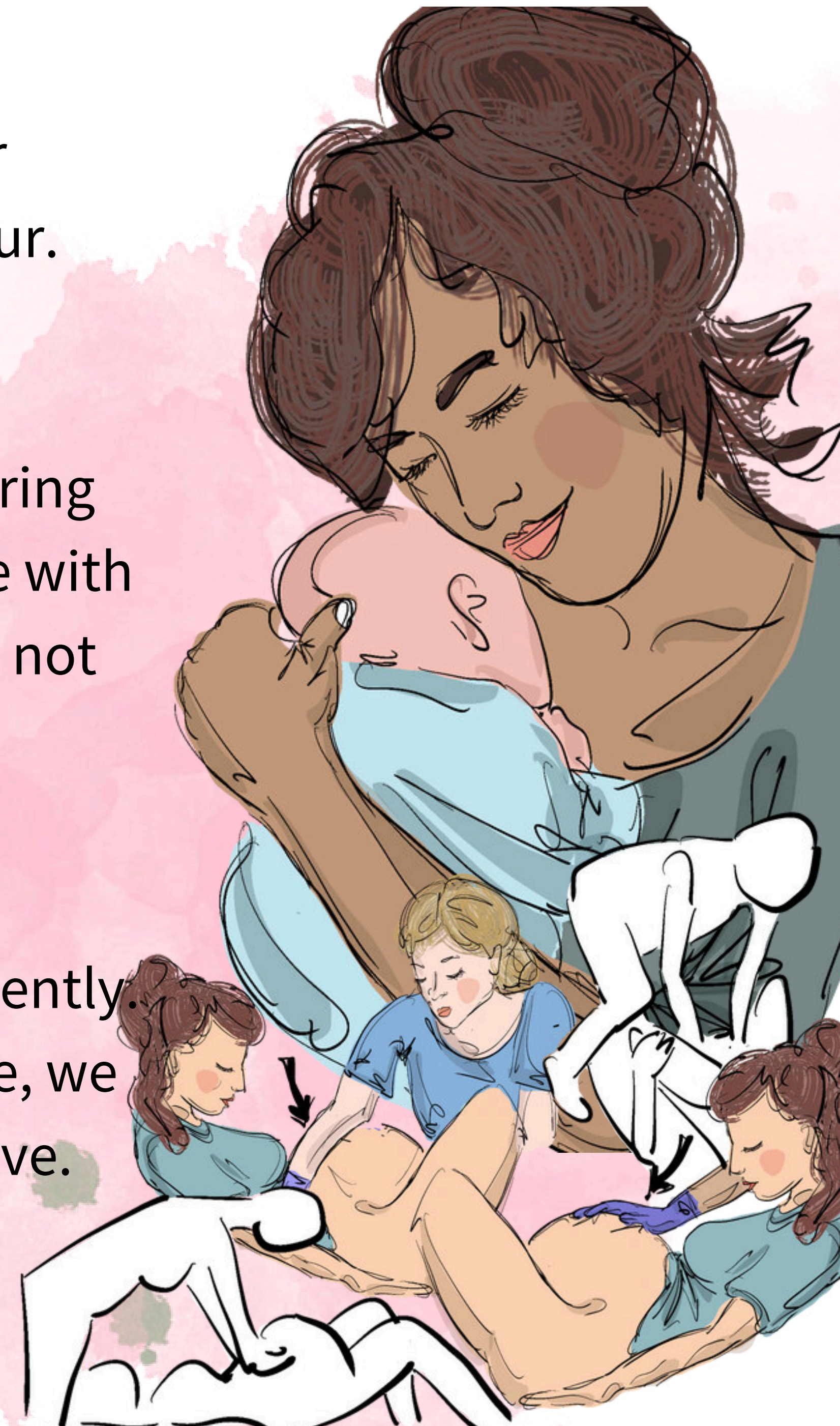

## Interview guide “Experience of obstetric violence (OV)”

Introduction:

- As you already know, we are exploring obstetric violence in order to better understand its background. Before we continue, I would like to express that I am very sorry that you have experienced violence during the birth of your child/children. Should we touch on aspects during the interview that you do not wish to talk about, please let me know. We want you to feel comfortable and safe at all times. If you need a break, please let me know. We can also agree on a sign (possible signs: raise of hand, ...) to let me know that you need a break. One last important remark: Our research focus is on the birth experience, not on the post partum period or breastfeeding.
- As already discussed, I would like to record the interview and it will later be transcribed for the analysis. That way I don't have to take notes and can listen to you better. Nevertheless, I may note down a few key points from time to time so that I can ask follow-up questions later on.
- Your perspective is important to us, so I will not interrupt you initially. Instead, I will simply listen and possibly take some notes.
- Ask interviewee if there are any questions.

**Narrative prompt: I now ask you to think about the birth of your child/children and tell me about the violent aspects.**

| Possible follow-up questions depending on the narration | Notes / keywords for follow-up questions |
|---|---|
| • What did you experience as violence / perceive as violent?<br>• What exactly did you perceive as [violent / disrespectful / intimidating / accusatory] when [reference to the respective description]?<br>• What would have helped you here? / What would you have wished for in this situation? / What kind of needs did you have? | |
| **<u>Relationship level</u>**<br><br>• How was your relationship with the staff that cared for you? (obstetrician, midwife) / How did you feel in this relationship?<br>• What did the process of decision-making look like? / What was the communication like?<br>• To what extent were you able to let go? / To trust? / To feel safe? | |
| **<u>Experience of autonomy</u>**<br><br>**Role of the caring staff**<br>• To what extent did the staff encourage or inhibit you in your ability to deal with the situation?<br>• To what extent were you given the space to trust in your own physical sensations, gut feeling or intuition?<br><br>**Sense of self-efficacy**<br>• To what extent did you feel like you were in a position of being able to cope with the demands of your birth?<br>• To what extent were you able to contribute personal skills and strengths during your birth?<br><br>**Perceived control**<br>• To what extent did you feel like you could influence the birthing process?<br>• To what extent were statements that you made taken into account (e.g. questions, requests, complaints)? | |

**Narrative prompt: Now that we talked about your birth experience, I ask you to tell me about your thoughts and feelings during pregnancy when looking forward to the birth of your child.**

| Possible follow-up questions depending on the narration | Notes / keywords for follow-up questions |
|---|---|
| **<u>Wishes / hopes</u>**<br><br>• What kind of wishes / hopes did you have with regards to the birth of your child?<br>• How did you want to give birth? | |

| | |
|---|---|
| • Where there any agreements / procedures / specific conditions that were of importance to you?<br>• Were there aspects that you did not want to be part of your experience under any circumstance?<br>• Which (of the above) aspects were particularly important to you? | |
| **<u>Expectations</u>**<br><br>**Personalised treatment by the caring staff**<br>• What kind of wishes / hopes did you have with regards to how staff would treat you on a personal level?<br>• How important was it to you to follow the staff's recommendations?<br><br>**Participation in the decision-making processes**<br>• To what extent did you want to participate in decision-making?<br>• How important was it for you to make birth-related decisions yourself? (prior to and during the birth experience)<br>• What aspects did you want to actively influence yourself during the birth?<br>• What expectations did you have with regards to your own performance?<br>• To what extent did previous birth experiences influence your expectations of this birth? | |
| **<u>Fulfillment of expectations</u>**<br><br>• To what extent did the caring staff respond to your wishes and needs during your birth? | |

**Closing part**

| | |
|---|---|
| • What would you like to say to [person who used violence]? | |
| • Thank you. We have come to the end of this interview. Are there any other points in relation to "violence during childbirth" that you would like to address? | |

**Thank you for sharing your experience, your trust and for helping to improve obstetric care.**

## Interview guide “Experience of fundal pressure during childbirth”

Introduction:

- As you already know, we are interested in how women experience fundal pressure during childbirth. The experiences differ greatly. Thank you for your willingness to share your experience and your point of view. Should we touch on aspects during the interview that you do not wish to talk about, I would ask you to please let me know. We want you to feel comfortable and safe at all times. If you need a break, please let me know. We can also agree on a sign (possible signs: raise of hand, ...) to let me know that you need a break. One last important remark: Our research focus is on the birth experience, not on the post partum period or breastfeeding.
- As already discussed, I would like to record the interview and it will later be transcribed for the analysis. That way I don't have to take notes and can listen to you better. Nevertheless, I may note down a few key points from time to time so that I can ask follow-up questions later on.
- Your perspective is important to us, so I will not interrupt you initially. Instead, I will simply listen and possibly take some notes.
- Ask interviewee if there are any questions.

**Narrative prompt: I now ask you to think about the birth of your child/children and tell me about the part of the birth in which the fundal pressure was applied.**

| Possible follow-up questions depending on the narration | Notes / keywords for follow-up questions |
|---|---|
| • What happened exactly?<br>• How was fundal pressure applied?<br>• How did you feel during the intervention?<br>  o Physically<br>  o Emotionally | |
| **<u>Own explanations</u>**<br>• What exactly did you perceive as [helpful/ pleasant or violent/ negative] in relation to [reference to the respective description]?<br><br>**<u>Negative aspects</u>**<br>• What would have helped you here? / What would you have wished for in this situation? / What should the caring staff have done differently in our opinion? | |
| **<u>Relationship level</u>**<br><br>**Communication and decision-making**<br>• How did the application of fundal pressure come about?<br>• How was the decision to exert fundal pressure made?<br>• To what extent were you involved in the decision-making?<br>• To what extent did you feel prepared for the application of fundal pressure?<br>• What did the communication regarding fundal pressure look like? What was said? How comprehensible was it to you?<br>• To what extent could you have stopped the executor of the fundal pressure if you had intended / tried to do so?<br>• If you did not want fundal pressure to be applied but did not express this, why did you not do so? | |
| **<u>Autonomy</u>**<br><br>**Perceived control**<br>• To what extent did the fundal pressure correspond to what you had expected/what was described to you beforehand?<br>• To what extent did you feel that you had control over the situation/the fundal pressure/to be able to stop or influence it?<br>• To what extent were your comments taken into account (e.g. questions, requests, complaints)? | |
| **<u>Relationship level</u>**<br><br>**Emotions**<br>• What was your relationship to the caring staff like? How did you feel with regards to this relationship?<br>• To what extent did you feel safe in this situation / trust the staff? | |

**Narrative prompt: Now that we talked about your fundal pressure experience, I ask you to tell me how you experienced the other stages of labour in the delivery room.**

| **Possible follow-up questions depending on the narration** | **Notes / keywords for follow-up questions** |
|---|---|
| • Did you perceive other aspects of your birth experience as [violent, bad, helpful, positive etc.]?<br>• What was your feeling before fundal pressure was applied? | |
| <u>**Relationship level**</u><br><br>**Emotions**<br>• To what extent did you feel like your wishes/hopes and needs were heard and seen?<br>• To what extent were statements that you made taken into account (e.g. questions, requests, complaints)? | |
| **Autonomy**<br><br>**Sense of self-efficacy**<br>• To what extent did you feel that you could manage your birth?<br>• To what extent did you feel like you could actively promote your birth?<br><br>**Perceived control**<br>• To what extent did you feel like you were in a position to influence your birth process? | |

**Narrative prompt: Now that we talked about your birth experience, I ask you to tell me about your thoughts and feelings during pregnancy when looking forward to the birth of your child.**

| **Possible follow-up questions depending on the narration** | **Notes / keywords for follow-up questions** |
|---|---|
| **Wishes / hopes**<br>• What kind of wishes / hopes did you have with regards to the birth of your child?<br>• How did you want to give birth?<br>• Where there any agreements / procedures / specific conditions that were of importance to you?<br>• Were there aspects that you did not want to be part of your experience under any circumstance?<br>• Which (of the above) aspects were particularly important to you?<br>• | |
| <u>**Expectations**</u><br><br>**Personalised treatment by the caring staff**<br>• What kind of wishes / hopes did you have with regards to how staff would treat you on a personal level?<br>• How important was it to you to follow the staff's recommendations? | |

| | |
|---|---|
| **Participation in the decision-making processes**<br>• To what extent did you want to participate in decision-making?<br>• How important was it for you to make birth-related decisions yourself? (prior to and during the birth experience)<br>• What aspects did you want to actively influence yourself during the birth?<br>• What expectations did you have with regards to your own performance?<br>• To what extent did previous birth experiences influence your expectations of this birth? | |
| **<u>Fulfillment of expectations</u>**<br><br>• To what extent did the caring staff respond to your wishes and needs during your birth? | |

**Closing part**

| | |
|---|---|
| • What would you like to say to the person/people that cared for you during birth?<br>• Thank you. We have come to the end of this interview. Are there any other points in relation to the fundal pressure and/or the birth of your child that you would like to address? | |

**Thank you for sharing your experience, your trust and for helping to improve obstetric care.**
